# Supplementary material for: Expanded Access Programme for the use of tecovirimat for the treatment of monkeypox infection: A study protocol for an Expanded Access Programme
Source: PLoS One. 2024 May 9;19(5):e0278957. doi: 10.1371/journal.pone.0278957 (PMC11081255; doi:10.1371/journal.pone.0278957)
Supplement: S1 Table — (DOC) [file pone.0278957.s001.doc]

**TECOVIRIMAT – CASE REPORT FORM**

| **INCLUSION** |
| --- |

| **RECRUITMENT** | |
| --- | --- |
| **Patient ID** | Site  : [___][___] – Patient  : [___][___][___] |
| **Date of evaluation** | [_D_][_D_]/[_M_][_M_]/[_Y_][_Y_] |
| **Date of appearance of first symptoms (lesions or fever)** | [_D_][_D_]/[_M_][_M_]/[_Y_][_Y_] |
| **Date of contact with an infected person or other source of infection** | [_D_][_D_]/[_M_][_M_]/[_Y_][_Y_]  ☐ Unknown |
| **Type of exposure** | ☐ Zoonotic exposure (contact with wildlife)  ☐ Contact with a member of the community  ☐ Contact with a health worker  ☐ Unknown |
| **Date of hospitalisation for suspected monkeypox** | [_D_][_D_]/[_M_][_M_]/[_Y_][_Y_] |
| **Name of site of identification** |  |

| **INCLUSION CRITERIA** | |
| --- | --- |
| **Is the patient a laboratory confirmed case of monkeypox or are they being managed as a presumptive case?** | ☐ The patient is a laboratory confirmed case of monkeypox  ☐ The patient is being managed as a presumptive case of monkeypox |
| **Weight ≥13 kg** | ☐ YES ☐ NO |
| **Is the patient taking repaglinide or midazolan?** | ☐ YES ☐ NO  If yes, the patient must be excluded from the study |
| **Does the patient have an intolerance to galactose, total lactase deficiency or glucose-galactose malabsorption?** | ☐ YES ☐ NO  If yes, the patient must be excluded from the study |
| **Has the patient (or their parent/guardian) provided written informed consent to be treated with tecovirimat and to participate in the study?** | ☐ YES ☐ NO |
| **Date of consent** | [_D_][_D_]/[_M_][_M_]/[_Y_][_Y_] |
| **Name of person who took consent** |  |

| | **DEMOGRAPHIC DATA** |  | | --- | --- | | **Sex at birth** | ☐Male ☐ Female | | **Age** | [___][___] years OR [___][___]months | | **Weight** | [______][___] (kg) | | **Is the patient pregnant ?** | ☐ YES ☐ NO ☐ N/A  If yes, number of weeks of gestation: [___][___] weeks | | **Is the patient breastfeeding?** | ☐ YES ☐ NO ☐ N/A |  | **COMORBIDITIES** |  | | --- | --- | | **hronic heart disease, including congenital heart disease** | ☐YES ☐NO ☐Unknown  If yes, specify : _______________________________________ | | **Chronic respiratory disease (e.g. asthma, COPD)** | ☐YES ☐NO ☐Unknown  If yes, specify : _______________________________________ | | **Diabetes (and type of diabetes)** | ☐YES Type I  ☐YES Type II  ☐NO  ☐Unknown | | **Chronic kidney disease** | ☐YES ☐NO ☐Unknown  If yes, specify : _______________________________________ | | **Moderate or severe liver disease** | ☐YES ☐NO ☐Unknown  If yes, specify : _______________________________________ | | **Chronic neurological disorder** | ☐YES ☐NO ☐Unknown  If yes, specify : _______________________________________ | | **Malignant neoplasm** | ☐YES ☐NO ☐Unknown  If yes, specify : _______________________________________ | | **Chronic haematological disease** | ☐YES ☐NO ☐Unknown  If yes, specify : _______________________________________ | | **Active sexually transmitted infection** | ☐YES  If yes, specify : _______________________________________  ☐NO  ☐Unknown | | **HIV/AIDS** | ☐YES – receiving antiretroviral treatment  ☐YES – not receiving antiretroviral treatment  ☐NO  ☐Unknown | | **Malnutrition** | ☐YES ☐NO ☐Unknown | |
| --- | --- | --- | --- | --- | --- | --- | --- | --- | --- | --- | --- | --- | --- | --- | --- | --- | --- | --- | --- | --- | --- | --- | --- | --- | --- | --- | --- | --- | --- | --- | --- | --- | --- | --- | --- | --- |
| | **CONCOMITANT MEDICATION** | | | --- | --- | | **HIV PREP/PEP** | ☐YES ☐NO ☐Unknown | | **Corticosteroids** | ☐YES ☐NO ☐Unknown | | **Other immunosuppressants** | ☐YES  If yes, specify : _______________________________________  ☐NO  ☐Unknown | |

**CLINICAL EVALUATION (to be completed at every study visit)**

| **SIGNS AND SYMPTOMS** | |
| --- | --- |
| **Date of evaluation :** [_D_][_D_]/[_M_][_M_]/[_Y_][_Y_] | |
| **Fever** | ☐YES ☐NO ☐Unknown Temperature: [__][__] . [__] |
| **Keratitis** | ☐YES ☐NO ☐Unknown |
| **Cough** | ☐YES ☐NO ☐Unknown |
| **Upper respiratory symptoms (sore throat, runny nose)** | ☐YES ☐NO ☐Unknown  If yes, specify ___________________ |
| **Lower respiratory symptoms (productive cough, wheezing, respiratory distress)** | ☐YES ☐NO ☐Unknown  If yes, specify ___________________ |
| **Lymphadenopathy** | ☐YES ☐NO ☐Unknown |
| **Nausea/vomiting** | ☐YES ☐NO ☐Unknown |
| **Diarrhoea** | ☐YES ☐NO ☐Unknown |
| **Headache** | ☐YES ☐NO ☐Unknown |
| **Encephalitis** | ☐YES ☐NO ☐Unknown |
| **Ocular complications** | ☐YES ☐NO ☐Unknown |
| **Pharyngitis** | ☐YES ☐NO ☐Unknown |
| **Deep tissue abscesses** | ☐YES ☐NO ☐Unknown |
| **Rectitis** | ☐YES ☐NO ☐Unknown |
| **Confirmed bacterial infection** | ☐YES ☐NO ☐Unknown  If YES, does the patient have any of the following infections :  ☐ Folliculitis/cellulitis  ☐Pneumonia  ☐Gastroenteritis  ☐CNS infection  ☐Bacteremia  ☐ Urinary tract infection  ☐Other, specify :___________________________________________ |
| **Muscular pain** | ☐YES ☐NO ☐Unknown |
| **Joint pain :** | ☐YES ☐NO ☐Unknown |
| **Seizures** | ☐YES ☐NO ☐Unknown |

| **LESION EVALUATION** | | |
| --- | --- | --- |
| **How many lesions does the patient have today?** | ☐ None  ☐ 1-5  ☐ 6-25  ☐ 26-100  ☐ 101-300  ☐ 301-500  ☐ >500 |  |
| **Have new lesions appeared in the last 24h?** | ☐YES ☐NO |  |

| **Are the following types of lesions present on the body today ?** | There are no lesions: all scabs have fallen off and the skin is intact | ☐YES ☐NO  If the answer is NO, please answer the following questions: | | |
| --- | --- | --- | --- | --- |
|  | Vesicule (active) | ☐YES ☐NO | | |
|  | Pustule (active) | ☐YES ☐NO | | |
|  | Ulcerated lesion (active) | ☐YES ☐NO | | |
|  | Haemorrhagic/ bleeding lesion (active) | ☐YES ☐NO | | |
|  | Mature lesion in the form of a crust (inactive) | ☐YES ☐NO | | |
| **Are there any active lesions in the following areas of the body ?** | Head, face or neck | ☐YES ☐NO | Genitals | ☐YES ☐NO |
|  | Arms or hands | ☐YES ☐NO | Inside of mouth | ☐YES ☐NO |
|  | Legs or feet | ☐YES ☐NO | Torso | ☐YES ☐NO |
|  | Other | ☐YES ☐NO Specify:__________________________________________ | | |
| **Lesion pain** | ☐YES ☐NO  If yes, level of pain : ☐ Low ☐ Medium ☐High | | | |
| **Lesion complications** | ☐YES ☐NO  If yes, please describe the complications: ___________________________________________  ____________________________________________________________________________  ____________________________________________________________________________  ____________________________________________________________________________ | | | |

| **VITAL SIGNS** | |
| --- | --- |
| **Respiratory rate** | [___][___]/min |
| **Pulse** | [___][___]/min |
| **Blood pressure** | Systolic [___] [___][___]/mmHg Diastolic [___][___]/mmHg |
| **Oxygen saturation** | [___][___]% ☐ Room air ☐ Oxygen therapy [___][___]L/min |

**TREATMENT INFORMATION (to be completed from D1 to D14)**

| **TREATMENT** | | |
| --- | --- | --- |
| **Tecovirimat (morning dose)** | ☐YES ☐NO ☐Unknown | Dose ____mg Time [_H_][_H_]/[_M_][_M_] |
|  | If the dose was forgotten modified or refused, state reason : _________________________ | |
| **Tecovirimat (evening dose)** | ☐YES ☐NO ☐Unknown | Dose ____mg Time [_H_][_H_]/[_M_][_M_] |
|  | If the dose was forgotten modified or refused, state reason : _________________________ | |
| **Paracetamol** | ☐YES ☐NO ☐Unknown | |
| **NSAID** | ☐YES ☐NO ☐Unknown | |
| **Intravenous fluids** | ☐YES ☐NO ☐Unknown | |

**OUTCOME ASSESSMENT (to be completed at D14 and D28 for all patients; and at D21 for patients who were still positive at D14)**

| **Date of evaluation** | [_D_][_D_]/[_M_][_M_]/[_Y_][_Y_] |
| --- | --- |
| **Has the patient followed the treatment according to the protocol? (D14 only)** | ☐ YES  ☐ NO  If no, state reason why:__________________________________________________________ |
| **Patient outcome** | ☐ The lesions are completely resolved and no serious complications remain  ☐ There are no more active lesions and no serious complications remain  ☐ One or more lesions are active and no serious complications remain  ☐ Serious complication and/or prolonged hospitalisation due to monkeypox  Specify the complication : _______________________  Date complication started : [_J_][_J_]/[_M_][_M_]/[_A_][_A_]  ☐ Death  Date of death : [_J_][_J_]/[_M_][_M_]/[_A_][_A_]  Cause of death : _______________________  ☐ Early withdrawal  ☐ Loss to follow-up  Reason for the loss to follow-up : _______________________ |

| **ADVERSE EVENT LOG** | | | | |
| --- | --- | --- | --- | --- |
| **Event name** | **Severity** | **Start date** | **End date** | **Outcome** |
|  | ☐ 1 - Mild  ☐ 2 - Moderate  ☐ 3 - Severe  ☐ 4 - Life-threatening  ☐ 5 - Death | [_D_][_D_]/[_M_][_M_]/[_Y_][_Y_] | [_D_][_D_]/[_M_][_M_]/[_Y_][_Y_] | ☐ Resolved without sequalae  ☐ Resolved with sequelae  ☐ Ongoing or not resolved  ☐ Death  ☐ Unknown |
|  | ☐ 1 - Mild  ☐ 2 - Moderate  ☐ 3 - Severe  ☐ 4 - Life-threatening  ☐ 5 - Death | [_D_][_D_]/[_M_][_M_]/[_Y_][_Y_] | [_D_][_D_]/[_M_][_M_]/[_Y_][_Y_] | ☐ Resolved without sequalae  ☐ Resolved with sequelae  ☐ Ongoing or not resolved  ☐ Death  ☐ Unknown |
|  | ☐ 1 - Mild  ☐ 2 - Moderate  ☐ 3 - Severe  ☐ 4 - Life-threatening  ☐ 5 - Death | [_D_][_D_]/[_M_][_M_]/[_Y_][_Y_] | [_D_][_D_]/[_M_][_M_]/[_Y_][_Y_] | ☐ Resolved without sequalae  ☐ Resolved with sequelae  ☐ Ongoing or not resolved  ☐ Death  ☐ Unknown |
|  | ☐ 1 - Mild  ☐ 2 - Moderate  ☐ 3 - Severe  ☐ 4 - Life-threatening  ☐ 5 - Death | [_D_][_D_]/[_M_][_M_]/[_Y_][_Y_] | [_D_][_D_]/[_M_][_M_]/[_Y_][_Y_] | ☐ Resolved without sequalae  ☐ Resolved with sequelae  ☐ Ongoing or not resolved  ☐ Death  ☐ Unknown |
|  | ☐ 1 - Mild  ☐ 2 - Moderate  ☐ 3 - Severe  ☐ 4 - Life-threatening  ☐ 5 - Death | [_D_][_D_]/[_M_][_M_]/[_Y_][_Y_] | [_D_][_D_]/[_M_][_M_]/[_Y_][_Y_] | ☐ Resolved without sequalae  ☐ Resolved with sequelae  ☐ Ongoing or not resolved  ☐ Death  ☐ Unknown |

Note: If the event 1) resulted in death, 2) was life threatening, 3) resulted in re-hospitalization or prolonged hospitalization, 4) resulted in disability or incapacity, 5) resulted in a birth defect, 6) or was otherwise medically significant, please complete the Serious Adverse Event Form. **Print this page as many times as necessary**

**WITHDRAWAL FORM**

| **Date of withdrawal** | [_D_][_D_]/[_M_][_M_]/[_Y_][_Y_] |
| --- | --- |
| **Reason for withdrawal** | ☐ Presumptive case who later received a negative PCR test for monkeypox  ☐ Intolerable adverse event  ☐ Patient was unable to comply with the protocol procedures  ☐ Other clinical decision, specify : __________________________  ☐ Participant decision, specify : __________________________ |
| **Did the participant withdraw from active participation in the study?** | ☐ YES  ☐ NO |
| **Has the participant withdrawn consent for the use of the data collected up to the point of discharge?** | ☐ YES  ☐ NO |
| **Did the participant withdraw consent for the use of the samples collected up to the time of discharge?** | ☐ YES  ☐ NO |

**PREGNANCY FOLLOW-UP FORM**

| **Has the mother consented to the monitoring of her pregnancy?** | ☐OUI ☐NON |
| --- | --- |
| **Date of pregnancy test :** | [_D_][_D_]/[_M_][_M_]/[_Y_][_Y_] |
| **Date of last period :** | [_D_][_D_]/[_M_][_M_]/[_Y_][_Y_] |
| **Expected due date :** | [_D_][_D_]/[_M_][_M_]/[_Y_][_Y_] |
| **Pregnancy outcome :** | ☐ Baby is in good health  ☐ Congenital malformations requiring admission to the neonatal unit  ☐ Induced abortion (by choice)  ☐ Therapeutic abortion (for medical reasons)  ☐ Miscarriage  ☐ Still birth  ☐ Neonatal death  ☐ [Maternal](https://www.linguee.com/french-english/translation/mortalité+maternelle.html) death |
| **Date of above outcome** | [_D_][_D_]/[_M_][_M_]/[_Y_][_Y_] |

| **TEST RESULTS** | | | | | | | |
| --- | --- | --- | --- | --- | --- | --- | --- |
|  | **Baseline** | **Day 1** | **Day 4** | **Day 8** | **Day 14** | **Day 21** ☐ N/A | **Day 28** |
| PCR Blood | Date of sample: [__][__]/[__][__]/[__][__]  ☐ Positive  ☐ Negative  ☐ Equivocal  ☐ Test not done  CT value: [__][__].[__] | Date of sample: [__][__]/[__][__]/[__][__]  ☐ Positive  ☐ Negative  ☐ Equivocal  ☐ Test not done  CT value: [__][__].[__] | Date of sample: [__][__]/[__][__]/[__][__]  ☐ Positive  ☐ Negative  ☐ Equivocal  ☐ Test not done  CT value: [__][__].[__] | Date of sample: [__][__]/[__][__]/[__][__]  ☐ Positive  ☐ Negative  ☐ Equivocal  ☐ Test not done  CT value: [__][__].[__] | Date of sample: [__][__]/[__][__]/[__][__]  ☐ Positive  ☐ Negative  ☐ Equivocal  ☐ Test not done  CT value: [__][__].[__] | Date of sample: [__][__]/[__][__]/[__][__]  ☐ Positive  ☐ Negative  ☐ Equivocal  ☐ Test not done  CT value: [__][__].[__] | Date of sample: [__][__]/[__][__]/[__][__]  ☐ Positive  ☐ Negative  ☐ Equivocal  ☐ Test not done  CT value: [__][__].[__] |
| PCR Lesion #1 | Date of sample: [__][__]/[__][__]/[__][__]  ☐ Positive  ☐ Negative  ☐ Equivocal  ☐ Test not done  CT value: [__][__].[__] | Date of sample: [__][__]/[__][__]/[__][__]  ☐ Positive  ☐ Negative  ☐ Equivocal  ☐ Test not done  CT value: [__][__].[__] | Date of sample: [__][__]/[__][__]/[__][__]  ☐ Positive  ☐ Negative  ☐ Equivocal  ☐ Test not done  CT value: [__][__].[__] | Date of sample: [__][__]/[__][__]/[__][__]  ☐ Positive  ☐ Negative  ☐ Equivocal  ☐ Test not done  CT value: [__][__].[__] | Date of sample: [__][__]/[__][__]/[__][__]  ☐ Positive  ☐ Negative  ☐ Equivocal  ☐ Test not done  CT value: [__][__].[__] | Date of sample: [__][__]/[__][__]/[__][__]  ☐ Positive  ☐ Negative  ☐ Equivocal  ☐ Test not done  CT value: [__][__].[__] | Date of sample: [__][__]/[__][__]/[__][__]  ☐ Positive  ☐ Negative  ☐ Equivocal  ☐ Test not done  CT value: [__][__].[__] |
| PCR Lesion #2 | Date of sample: [__][__]/[__][__]/[__][__]  ☐ Positive  ☐ Negative  ☐ Equivocal  ☐ Test not done  CT value: [__][__].[__] | Date of sample: [__][__]/[__][__]/[__][__]  ☐ Positive  ☐ Negative  ☐ Equivocal  ☐ Test not done  CT value: [__][__].[__] | Date of sample: [__][__]/[__][__]/[__][__]  ☐ Positive  ☐ Negative  ☐ Equivocal  ☐ Test not done  CT value: [__][__].[__] | Date of sample: [__][__]/[__][__]/[__][__]  ☐ Positive  ☐ Negative  ☐ Equivocal  ☐ Test not done  CT value: [__][__].[__] | Date of sample: [__][__]/[__][__]/[__][__]  ☐ Positive  ☐ Negative  ☐ Equivocal  ☐ Test not done  CT value: [__][__].[__] | Date of sample: [__][__]/[__][__]/[__][__]  ☐ Positive  ☐ Negative  ☐ Equivocal  ☐ Test not done  CT value: [__][__].[__] | Date of sample: [__][__]/[__][__]/[__][__]  ☐ Positive  ☐ Negative  ☐ Equivocal  ☐ Test not done  CT value: [__][__].[__] |
| PCR Throat | Date of sample: [__][__]/[__][__]/[__][__]  ☐ Positive  ☐ Negative  ☐ Equivocal  ☐ Test not done  CT value: [__][__].[__] | Date of sample: [__][__]/[__][__]/[__][__]  ☐ Positive  ☐ Negative  ☐ Equivocal  ☐ Test not done  CT value: [__][__].[__] | Date of sample: [__][__]/[__][__]/[__][__]  ☐ Positive  ☐ Negative  ☐ Equivocal  ☐ Test not done  CT value: [__][__].[__] | Date of sample: [__][__]/[__][__]/[__][__]  ☐ Positive  ☐ Negative  ☐ Equivocal  ☐ Test not done  CT value: [__][__].[__] | Date of sample: [__][__]/[__][__]/[__][__]  ☐ Positive  ☐ Negative  ☐ Equivocal  ☐ Test not done  CT value: [__][__].[__] | Date of sample: [__][__]/[__][__]/[__][__]  ☐ Positive  ☐ Negative  ☐ Equivocal  ☐ Test not done  CT value: [__][__].[__] | Date of sample: [__][__]/[__][__]/[__][__]  ☐ Positive  ☐ Negative  ☐ Equivocal  ☐ Test not done  CT value: [__][__].[__] |
| Test de grossesse | Date of test : [__][__]/[__][__]/ [__][__]  ☐ Positive  ☐ Negative  ☐ Test not done |  | - | - | - | - | - |
| Malaria RDT | Date of test : [__][__]/[__][__]/ [__][__]  ☐ Positive  ☐ Negative  ☐ Test not done |  | - | - | - | - | - |
| HIV test RDT | Date of test : [__][__]/[__][__]/ [__][__]  ☐ Positive  ☐ Negative  ☐ Test not done | - | - | - | - | - | - |
| HIV test (lab) | Date of test : [__][__]/[__][__]/ [__][__]  ☐ Positive  ☐ Negative  ☐ Test not done | - | - | - | - | - | - |

| **ADDITIONAL TESTS** |
| --- |

| **Date of test :** | [_D_][_D_]/[_M_][_M_]/[_Y_][_Y_] |
| --- | --- |
| **Corresponding study day :**  *e.g. : D1, D2, D3 etc.* |  |
| **Type of test :** |  |
| **Reason for the test :** |  |
| **Test result :**  *If you are reporting a numerical result, please specify the units* |  |

**DECLARATION OF APPROVAL FROM THE PRINCIPAL INVESTIGATOR**

| I have reviewed this CRF and confirm that, to the best of my knowledge, it accurately reflects the information obtained for this participant. The information was entered either by myself or by a person under my supervision who was assigned according to the delegation of authority register. |
| --- |
| **Name :** _______________________  **Signature :** ____________________  **Date :** [_D_][_D_]/[_M_][_M_]/[_Y_][_Y_] |
