# Supplementary material for: Expanded Access Programme for the use of tecovirimat for the treatment of monkeypox infection: A study protocol for an Expanded Access Programme
Source: PLoS One. 2024 May 9;19(5):e0278957. doi: 10.1371/journal.pone.0278957 (PMC11081255; doi:10.1371/journal.pone.0278957)
Supplement: S2 Text — (DOCX) [file pone.0278957.s003.docx]

**Patient Information Sheet: Treatment of monkeypox with tecovirimat**

**Version 3.0 - 9 August 2022**

**<INSERT INVESTIGATOR DETAILS>**

**Treatment location**: [Add name and address of health centre]

We are going to give you some information about a medicine that might help you get better. The medicine is called tecovirimat.

**Why are you asking me to take tecovirimat?**

You have an illness called Monkeypox. Monkeypox is a serious disease that makes you sick with fever and chills, fatigue, aching of the head, muscles, and back. It caused lymph nodes to swell, and your skin to be scattered with lesions like macules, vesicles, pustules or scabs. This may last for 2−4 weeks. In some cases, monkeypox can cause long lasting effects even if you have recovered from the disease and in a minority of cases may even be fatal.

Tecovirimat has recently been approved to treat monkeypox in some countries, but we would like to learn more about using tecovirimat to treat monkeypox. We think this it might help you get rid of Monkeypox more quickly and get better sooner.

However, we cannot give you this medicine unless you say you want to have it. This is because tecovirimat is but not used in routine care for Monkeypox in the Central African Republic but is used in routine care for a disease similar to Monkeypox, so we think tecovirimat may also help people like you with Monkeypox. However, we don’t know that it will definitely help you because there haven’t been enough studies yet to see if it helps people get better. But we do know that it is safe for people to take and it shouldn’t make you feel worse. In some cases, like this one, we are allowed to offer you a new medicine, but only if you agree too.

The purpose of this form is to help you understand how tecovirimat works and to help you to decide whether you want us to use it to treat you.

You do not have to agree to this treatment if you don’t feel like it. Treatment is completely voluntary.

Please read this information carefully, or someone can read it to you. Please ask questions if there is anything you do not understand or would like to know more about.

**What will happen if I decide to take tecovirimat?**

Your doctor will ask you about your health and other medicines that you might be taking. You will be offered a HIV test and, if you are female, you will be offered a pregnancy test. These tests are voluntary and you do not have to take them if you don’t want to.

If your doctor thinks it is safe for you to participate, you will stay at the hospital and take the medicine for 14 days. Your doctor will weigh you and ask you to take the medicine with some liquid or food. Tecovirimat is a tablet that you swallow. While you are at the hospital, your doctor will ask you how you are feeling, examine you and perform some tests on your blood, throat and rashes.

We will take blood samples, lesions and throat swabs from you at 6 time points. Samples and swabs will be taken before you start taking tecovirimat and further samples will be taken on days 4, 8, 14 and 21 (if required) of your hospital stay. Final samples and swabs will be taken about one month after you first started taking tecovirimat. These samples and swabs will be used to look at how your body reacts to the medicine. This will help us to decide whether we can use this medicine for other people in the future.

We will collect some information about you and your health while you are taking tecovirimat because we want to learn whether this could be a good treatment for other people with monkeypox too. This information will be collected before you start taking the medicine and until about one month after you first started taking the medicine. We would also like to take some photos of the rashes or blisters on your skin that have been caused by monkeypox.

Your doctor may also stop giving you tecovirimat at any time if they feel this is in your best interest.

If you don’t want to take tecovirimat, you don’t have to. Remember, taking tecovirimat is up to you and no one will be upset if you don’t want to or if you change your mind later and want to stop. We will give you the best available care whether you take tecovirimat or not. You can ask any questions that you have about tecovirimat. If you have a question later that you didn’t think of now, you can ask your doctor next time.

**Are there any risks or disadvantages to me when taking part?**

Because tecovirimat has only recently started to be used to treat Monkeypox, there may be risks that we do not know about at this time. The side-effects we know about are:

- Your head might hurt
- Your stomach might hurt
- You might feel sick (feel like vomiting) or you might actually vomit.

There may be other side-effects that we don’t know about yet and we don’t know what effect tecovirimat has in pregnancy. You must ensure that you contact your doctor if you have any side effects so they can tell you what to do.

You may also feel a small scratch when the blood samples are taken.

**Are there any benefits to me taking part?**

We hope that tecovirimat will help you get better, but don’t know that it definitely will. We hope that the information we collect from people who decide to take the medicine will help other people in the future.

**What are the alternative treatment options?**

Currently there is no other treatment that can cure Monkeypox. That’s why we are offering tecovirimat to you. However, we will give you the best care we can if you decide not to take tecovirimat. You may choose not to receive tecovirimat now or change your mind later. Your doctor may also stop the treatment if they feel it is in your best interest not to continue this treatment. Your decision will not affect the quality of care you receive.

**How much will tecovirimat treatment cost?**

Tecovirimat will be given to you free of charge.

**What if something goes wrong?**

It is important that you immediately tell your doctor if you think that you have been injured because of this treatment. You can tell your doctor in person or call him/her at the numbers listed at the top of this form.

**What will happen to my information?**

If you agree to take tecovirimat, your consent form will be held at Institut Pasteur de Bangui for a minimum of 3 years and information about your health will be kept in your medical record ind on a database with us. This information will be kept for at least 25 years.

All information about you will be kept confidential. Your name will not be used on your blood tests, data or in any reports about tecovirimat. Instead we will use a ‘subject number’ – a unique number given to you. All the data we collect about you and your treatment with tecovirimat will be recorded on to a paper form and then entered into a database.

The University of Oxford is the ‘data controller’, so we look after your personal data. We will not share any information that could identify you.  Because of the special conditions under which we can give you this treatment that we explained before, we also must share some of the information about your illness with the company (SIGA Technologies Inc) who make the medicine and with health authorities in the Central Africa Republic and other countries, specifically the FDA in the USA, but we will not share any information that could identify you.

The University will process your personal data for the purpose of the research we’ve described above.  Research is a task that is performed in the public interest, and your data will only be used in the ways we have told you about in this information sheet.

Further information about your rights with respect to your personal data is available from <http://www.admin.ox.ac.uk/councilsec/compliance/gdpr/individualrights/>

**What will happen to my samples?**

Your samples will be tested to see whether tecovirimat has helped you get better. Your samples will be labelled with your patient ID only (they will not contain your name or any other information that can identify you). Your samples will be stored at Institute Pasteur Bangui for 5 years*.*

**What if you find out important information while I’m taking tecovirimat?**

If we find out some important new information about tecovirimat that could affect you while you are having the treatment, we will immediately inform your doctor.

**What if I have a question?**

If you have any concerns or questions about your treatment, please contact your doctor on the numbers listed at the top of this form or talk to them when you are next at the hospital.

We will be happy to answer any questions or concerns you have at any time. You are also welcome to talk to family and friends, or ask someone else, before you agree to take part. If you have questions after you leave the treatment center, you can call your doctor. They can also give you other numbers for information about your rights as a participant.

**Who has given you permission to offer tecovirimat?**

The following committees have reviewed our protocol and given us permission to offer tecovirimat to patients with monkeypox:

- Oxford Tropical Research Ethics Committee at Oxford University in the United Kingdom
- Comité Scientifique Charge de la Validation des Protocoles d’Etudes et des Résultats de Recherche en Santé

***Informed Consent Form (ADULTS)***

**It is your choice if you want to participate or not. Read the following statements and if you agree, please initial the box next to the statements:**

- All of the information above is clear, I understand the risks and benefits of taking tecovirimat and that my questions were answered appropriately.
- Any of my leftover blood, lesion (rash) and throat samples can be stored and used for research on monkeypox in the future.
- I understand I can stop taking tecovirimat at any time and I will still receive the best available care at the treatment centre.
- I agree to take tecovirimat

Time and date that information was discussed. Time ___:___ Date ___/___/______

Patient name: __________________________________

Subject Identification Number: ___________________

Time and date of signature Time ____:____ Date ___/___/_____

| Patient signature:  x_____________________________________________________ |
| --- |

| I have followed the procedures to obtain informed consent. The patient (or their representative) has freely agreed to participate. Signature of person taking consent:  x____________________________ | Name of person obtaining consent:  x______________________________ |
| --- | --- |

If the person giving consent cannot read the consent form by themselves, a witness should be present to hear this form read accurately to the patient and ensure that the information is explained to them such that they can understand. The person signing below confirms that informed consent was freely given by the patient.

| Witness signature:  x___________________________ | Witness name:  x____________________________________ |
| --- | --- |

***Informed consent form (CHILDREN AND PROXY)***

**It is up to you to choose whether or not you want your relative or friend to receive tecovirimat. Read the following statements and if you agree, please initial the box next to the statements:**

- All the above information is clear, I understand the risks and benefits of the taking tecovirimat and my questions have been appropriately answered.
- Any leftover blood, lesion (rash) and throat samples can be stored and used for monkeypox research in the future.
- I understand that I can terminate the member's treatment at any time and that he or she will always receive the best care available at the treatment centre.
- I agree for my relative or friend to receive tecovirimat

The time and date on which this information was discussed. Time ___:___ Date ___/___/_________

Patient name: __________________________________ Subject Identification Number: ___________________

Time and date of signature Time ____:____ Date ___/___/_____

| Signature of the person giving consent:  __________________________ x | Name of the person giving consent:  _______________________ x | Relationship to the patient:  _________________________ x |
| --- | --- | --- |

| I followed the procedures to obtain informed consent. The patient (or his or her friend, relative or representative) has freely agreed to participate. Signature of the person taking consent:  ____________________________ x | Name of person obtaining consent:  ______________________________ x |
| --- | --- |

If the person giving consent cannot read the form by himself or herself, a witness should be present to hear the form accurately read to the patient and ensure that the information is explained to him or her so that he or she can understand. The person signing below confirms that informed consent has been freely given by the patient.

| Witness' signature:  ___________________________ x | Name of the witness:  ____________________________________ x |
| --- | --- |
