# Supplementary material for: Expanded Access Programme for the use of tecovirimat for the treatment of monkeypox infection: A study protocol for an Expanded Access Programme
Source: PLoS One. 2024 May 9;19(5):e0278957. doi: 10.1371/journal.pone.0278957 (PMC11081255; doi:10.1371/journal.pone.0278957)
Supplement: Attachment — Submitted filename: Response to reviewers.docx [file pone.0278957.s004.docx]

Date: 17-Feb-2023

Dear Editorial Team,

Re: PLOS ONE Decision: Revision required [PONE-D-22-32294]

Thank you for your feedback on our manuscript. We have responded to each of the reviewers’ comments in the Table below.

Please let us know if any further action is required.

Sincerely,

Josie Bourner

| **Reviewer comment** | **Author response** |
| --- | --- |
| Please ensure that your manuscript meets PLOS ONE's style requirements, including those for file naming. | We have amended the manuscript to align with the journal requirements. |
| We note that you have referenced "Institut Pasteur de Bangui. Unpublished data from the monkeypox reference laboratory at Institut Pasteur de Bangui. 2022." which has currently not yet been accepted for publication. Please remove this from your References and amend this to state in the body of your manuscript: (ie “Bewick et al. [Unpublished]”) as detailed online in our guide for authors | We have amended this reference. |
| We note that the grant information you provided in the ‘Funding Information’ and ‘Financial Disclosure’ sections do not match. | The funding information provided in the manuscript is correct: SIGA donated 100 treatments for use under the EAP. The EAP was financed with core funds of IPB, financial support to IPB from SIGA, the University of Oxford under the UK Foreign, Commonwealth and Development Office and Wellcome (215091/Z/18/Z) and the Bill & Melinda Gates Foundation (OPP1209135).  Note: there are no grant award numbers for SIGA’s donation to of tecovirimat to IPB, core funds of IPB, or the financial support of SIGA to IPB. |
| Please include your full ethics statement in the ‘Methods’ section of your manuscript file. | This information has been moved to the Methods section. |
| We note that you have stated that you will provide repository information for your data at acceptance. Should your manuscript be accepted for publication, we will hold it until you provide the relevant accession numbers or DOIs necessary to access your data. If you wish to make changes to your Data Availability statement, please describe these changes in your cover letter and we will update your Data Availability statement to reflect the information you provide | Please could you amend our Data Availability statement to indicate that there is no data associated with this manuscript. |
| Please ensure that you refer to Figure 1 in your text as, if accepted, production will need this reference to link the reader to the figure. | Figure 1 has been amended to Table 4 and has now been referenced in the text. |
| We note you have included a table to which you do not refer in the text of your manuscript. Please ensure that you refer to Tables 1 and 2 in your text; if accepted, production will need this reference to link the reader to the Table | We have added a reference to Tables 1 and 2 in the text. |
| Please review your reference list to ensure that it is complete and correct. If you have cited papers that have been retracted, please include the rationale for doing so in the manuscript text, or remove these references and replace them with relevant current references. Any changes to the reference list should be mentioned in the rebuttal letter that accompanies your revised manuscript. If you need to cite a retracted article, indicate the article’s retracted status in the References list and also include a citation and full reference for the retraction notice. | We are unsure which retracted article you are referring to in the reference list. Please could you clarify?  We have moved the two unpublished data sources from IPB from the reference list and added them as citations in the text as per the manuscript guidelines (formerly references 4 and 14). |
| Page 19: “arel” is a spelling error | This has been amended to “will” |
| Indicate if adverse event reporting will be conducted according to a standardized method | We have added information about the collection of AEs and SAEs and a confirmation that our process complies with the EMA’s E6 guideline. |
| The statistical analysis plan should be specific. List the statistical methods that will used for summarizing the descriptive analyses, i.e., means, standard deviations, median, first and third quartiles, frequencies and percentages. Be sure to also state how the adverse events data will be summarized. Indicate how variables that are repeatedly measured will be summarized. Will any inferential statistical testing be conducted to compare changes in outcomes? | We have described in detail how we will present demographic information, baseline and post-baseline signs and symptoms, patient outcomes and any AEs and SAEs that may be collected during the study.  We have also confirmed that no inferential statistical testing will be used due to the nature of this study. |
| To assist in the review process, add line numbering to the document | We have added line numbering to the manuscript. |
